# Whole-genome profiling and shotgun sequencing delivers an anchored, gene-decorated, physical map assembly of bread wheat chromosome 6A

**DOI:** 10.1111/tpj.12550

**Published:** 2014-05-09

**Authors:** Naser Poursarebani, Thomas Nussbaumer, Hana Šimková, Jan Šafář, Hanneke Witsenboer, Jan van Oeveren, Jaroslav Doležel, Klaus FX Mayer, Nils Stein, Thorsten Schnurbusch

**Affiliations:** 1 Leibniz Institute of Plant Genetics and Crop Plant Research (IPK)Corrensstr. 3, D-06466, Stadt Seeland (OT) Gatersleben, Germany; 2 MIPS/IBIS German Research Center for Environmental HealthD-85764, Neuherberg, Germany; 3 Institute of Experimental Botany, Centre of the Region Haná for Biotechnological and Agricultural ResearchCZ-78371, Olomouc, Czech Republic; 4 Keygene N.V.Agro Business Park 90, 6708 PW, Wageningen, The Netherlands

**Keywords:** bread wheat chromosome 6A, whole-genome profiling, linear topological contigs, anchored physical map, bacterial artificial chromosome contigs, technical advance

## Abstract

Bread wheat (*Triticum aestivum* L.) is the most important staple food crop for 35% of the world's population. International efforts are underway to facilitate an increase in wheat production, of which the International Wheat Genome Sequencing Consortium (IWGSC) plays an important role. As part of this effort, we have developed a sequence-based physical map of wheat chromosome 6A using whole-genome profiling (WGP™). The bacterial artificial chromosome (BAC) contig assembly tools fingerprinted contig (fpc) and linear topological contig (ltc) were used and their contig assemblies were compared. A detailed investigation of the contigs structure revealed that ltc created a highly robust assembly compared with those formed by fpc. The ltc assemblies contained 1217 contigs for the short arm and 1113 contigs for the long arm, with an L_50_ of 1 Mb. To facilitate *in silico* anchoring, WGP™ tags underlying BAC contigs were extended by wheat and wheat progenitor genome sequence information. Sequence data were used for *in silico* anchoring against genetic markers with known sequences, of which almost 79% of the physical map could be anchored. Moreover, the assigned sequence information led to the ‘decoration’ of the respective physical map with 3359 anchored genes. Thus, this robust and genetically anchored physical map will serve as a framework for the sequencing of wheat chromosome 6A, and is of immediate use for map-based isolation of agronomically important genes/quantitative trait loci located on this chromosome.

## Introduction

Bread wheat (*Triticum aestivum* L.) has been a constant staple food and major crop that has provided energy and protein for humankind for millennia. Today, it represents approximately 20% of all calories consumed by humans (http://www.fao.org). As the world population grows and the climate continuously changes, future generations may challenge the current food supplies, which could augment the demand for wheat (Ortiz *et al.*, 2008; Sommer *et al.*, 2013). Therefore, accelerating wheat breeding and production by understanding the molecular basis of phenotypic variation and exploiting genetic diversity to improve performance are crucial. In this respect, the sequencing of the entire bread wheat genome has been considered to be a critical step for achieving these goals (Eversole *et al.*, 2009).

Accessing complete chromosomal sequences and the gene repertoire of hexaploid wheat (2*n* = 6*x* = 42) is quickly becoming a necessary but daunting task because of its large genome size (∼17 Gb/1C), which has resulted from successive hybridization events of three diploid grasses with large and structurally similar genomes (A, B and D), populated with more than 80% of repetitive elements (Flavell *et al.*, 1977). To overcome these challenges, a strategy has been identified that aims to break down genomic analyses into manageable-sized tasks (i.e. individual chromosomes and/or chromosome arms). This strategy relies on flow-sorting of individual chromosome arms used for construction of BAC libraries and also for next-generation sequencing (Doležel *et al*., 2007, 2014). Moreover, together with the advent of physical map building using whole-genome profiling (WGP™; van Oeveren *et al.*, 2011), chromosome flow cytometry represents an important technological development that provides easier access to large and polyploid genomes (Doležel *et al*., 2014).

Whole-genome profiling (WGP™) produces sequence tags at terminal ends of enzymatic restriction fragments from individual bacterial artificial chromosome (BAC) clones using a short-read ‘Next Generation’ or ‘High Throughput’ sequencing device (van Oeveren *et al.*, 2011). Identification of BAC overlaps by pairwise WGP™ tag comparisons allows for the assembly of BAC clones into contigs using the fingerprinted contigs (fpc; Soderlund *et al.*, 1997) or linear topological contig (ltc) programs (Frenkel *et al.*, 2010). WGP™ is considered more robust, less laborious and more efficient at building physical contigs than conventional high-information content fingerprinting (HICF; Luo *et al.*, 2003; Philippe *et al.*, 2012). The WGP™ approach has been successfully used on a number of genomes (van Oeveren *et al.*, 2011; TTGC, 2012; Sierro *et al.*, 2013), and its application has also been demonstrated for BAC clone contig formation originating from only a small fraction of wheat chromosome 3B (Philippe *et al.*, 2012). Moreover, WGP™ short sequence tags connected to the sequence contigs obtained from whole-chromosome shotgun sequencing (WCS; or chromosome shotgun sequencing, CSS) may further expand the possibilities for using WGP™.

Substantially longer WCS contigs connected to BAC-associated WGP™ tags facilitate sequence homology searches against respective genetic markers with known sequences, thereby providing an *in silico*-anchored physical map. A physical map, aided by shotgun sequencing, can provide clear insight into the physically positioned and ordered gene repertoire before complete genomic sequences of the wheat chromosomes become available. Without a link to physical maps, individual wheat chromosome shotgun sequence data sets were previously used to estimate virtual gene order, composition and evolutionary chromosomal rearrangements (Vitulo *et al.*, 2011; Hernandez *et al.*, 2012; Akhunov *et al.*, 2013; Tanaka *et al.*, 2014). Such studies were mainly performed by an analysis of extensive long-range conserved synteny with reference grass genomes [*Oryza sativa* (rice), *Sorghum bicolor* and *Brachypodium distachyon*]. Similarly, a fivefold genome coverage-based sequence assembly of the entire bread wheat genome shotgun sequence was previously completed and analyzed (Brenchley *et al.*, 2012) using a similar comparative genomics-based approach that involved comparison with sequences of diploid ancestral and progenitor genomes. Although these reports represent significant achievements in wheat genome biology, a more accurate wheat reference genome that avoids assumptions made by comparative genomics requires the establishment of genetically anchored physical maps.

Genes assigned to a physical map significantly facilitate positional gene cloning efforts and detection of regulatory elements. Herein, we constructed a physical map of wheat chromosome 6A linked to the annotated gene sequence information. We report a high-resolution gene map of chromosome 6A based on DNA sequences obtained from flow-sorted chromosome arms and by using the WGP™-based physical map approach. BAC assembly was performed using ltc software, and the assembly robustness was compared with fpc. WCS contigs from hexaploid wheat chromosome 6A [International Wheat Genome Sequencing Consortium (IWGSC), http://www.wheatgenome.org], together with available sequences from wheat ancestral diploid genomes (Jia *et al.*, 2013; Ling *et al.*, 2013), were aligned to the 6A physical map. Overall, we describe the development of a powerful resource for 6A that facilitates the study of its assigned genes and quantitative trait loci (QTLs).

## Results

### BAC libraries of the 6A chromosome arms

Bacterial artificial chromosome libraries were constructed separately from short (6AS) and long (6AL) arms of chromosome 6A, which were purified separately by flow-cytometric sorting from two telosomic lines of wheat in which the arms originating from 6A of cv. Chinese Spring are stably maintained as telocentric chromosomes. Analysis of random samples of sorted chromosome fractions by fluorescence *in situ* hybridization (FISH) revealed an average purity of 89 and 86% for 6AS and 6AL, respectively. A total of 7.15* *million and 6* *million 6AS and 6AL arms were collected, which represented 4.91 and 4.53* *μg DNA, respectively. The DNA was used to construct chromosome arm-specific BAC libraries TaaCsp6AShA (6AS; 49* *152 clones, average insert size 125 Kb; *Hin*dIII) and TaaCsp6ALhA (6AL; 55* *296 clones, average insert size 123 Kb; *Hin*dIII). Considering the molecular size of 336 Mbp for 6AS and 369* *Mbp for 6AL (Šafář *et al*., 2010), as well as contamination from other chromosomes, the libraries represent ∼16 equivalents of the 6A arms (Table 1).

**Table 1:** Chromosome 6A arm-specific BAC libraries used to construct the physical maps

No. of BAC clone	Short arm (6AS; 336 Mb)^a^	Long arm (6AL; 369 Mb)^b^	Library coverage
Complete BAC library	49 152	55 296	∼16×
For WGP™	22 656	24 576	∼8×
As WGP™ output	19 289	18 660	∼7×
WGP™ output used for BAC assembly^b^	18 820	17 309	∼7× (6AS) & ∼5× (6AL)

WGP™, whole-genome profiling.

a Šafář *et al*. (2010).

b BACs containing <6 or >68 tags were not entered into the physical map assembly pipeline.

### BAC contig assembly of the 6A chromosome arms

Whole-genome profiling (WGP™) data were produced for both chromosome arms 6AS and 6AL (Table 2; Appendix S1). Only BAC clones containing 6–68 tags entered the BAC assembly pipelines. A total of 18 820 BAC clones and 109 570 unique WGP™ tags were used as input for BAC assembly of 6AS (Table 2). For 6AL, 17 309 BAC clones containing 108 700 unique WGP™ tags were used to build the physical map (Table 2). This delivered an average of 29.7 WGP™ tags per BAC for 6AS and 28.6 WGP™ tags per BAC for 6AL, by considering all BACs for a given WGP™ tag (Table 2). We used ltc and fpc tools for assembly. ltc has been shown to outperform fpc because it can build longer, better ordered and mo re robust contigs, compared with fpc, employing HICF-based BAC fingerprints (Breen *et al.*, 2013; Lucas *et al.*, 2013; Philippe *et al.*, 2013; Raats *et al.*, 2013). The same set of BACs and WGP™ tags were used for both fpc and ltc analyses.

**Table 2:** Overview of general whole-genome profiling (WGP™) parameters and sequence data processing

Parameter	6AS	6AL
Estimated size of chromosome	336 Mbp	369 Mbp
WGP™ tag length, incl. restriction site^a^	50 nt	50 nt
No. of BACs tested	22 656	24 576
Chromosome arm equivalent BACs tested	8.4	7.0
No. of high-quality reads produced (M)	99.8	165.4
No. of deconvolutable reads (M)	52.2	78.2
Percentage of deconvolutable reads (%)	52.30	47.30
No. of tagged BACs	19 289	18 660
No. of tagged BACs used in assembly	18 820	17 309
No. of unique WGP™ tags	109 611	108 811
No. of unique WGP™ tags used in assembly	109 570	108 700
Percentage of tagged BACs (%)	85	75.9
Average no. of WGP™ tags/BAC	29.7	28.6
Average no. of reads/tag	61.4	111.1

BAC, bacterial artificial chromosome.

b The enzyme combination applied was *Hind*III/*Mse*I for both libraries.

Automated ltc delivered 1217 contigs and 3136 singletons for 6AS and 1113 contigs and 2581 singletons for 6AL (Table 3; Appendices S2 and S3). All contigs contained at least two BAC clones. The 6AS average contig size was estimated to be 0.429* *Mb with an L_50_ contig size of 1* *Mb, whereas the 6AL physical map had an average of 0.488* *Mb and had an L_50_ contig size of 0.945* *Mb (Table 3). All contigs were validated for linear topology and represented as the net width value. Contigs with a width >1, which indicates the presence of clone(s) having no significant overlaps with clones from the selected minimal tiling path (MTP) of the contig, were considered questionable because their topological network representation contradicted the linear chromosomal structure. Almost 95% of the cumulative 6AS and 6AL contigs were initially linear, whereas the rest, having a width ≥2, were checked manually for linearity. In the latter group, nonlinearity was presumably caused by missing WGP™ tags in a particular single BAC. Thus, these contigs were kept as such and further break down was not performed. Using ltc, a total of 5139 (6AS) and 5621 (6AL) clones were selected for the MTP (Appendices S2 and S3). Another physical map was made by fpc (1e^−11^ final cut-off; Appendix S4), in which a total of 640 and 620 contigs for 6AS and 6AL were formed, respectively. A total of 5045 and 3560 BACs were sorted out as singletons for 6AS and 6AL, respectively (Table S1; Appendix S5).

**Table 3:** Chromosome 6A arm-separated assemblies via linear topological contigs (ltc), with at least two BAC clones

	6AS			6AL		
	All	ltc-specific contigs^a^	Contigs common to fpc (1e^−11^)^b^	All	ltc-specific contigs^a^	Contigs common to fpc (1e^−11^)^b^
Total no. of contigs (≥2 clones)	1217	539	678	1113	368	745
Singleton	3136	–	–	2581	–	–
Minimum Kb	42.3	42.3	98.7	47	47	65.8
Maximum Kb	9531.6	470	9531.6	3139.6	521.7	3139.6
Average Kb	428.526	131.033	665.029	487.887	123.336	667.961
L_50_ Kb	1090.4	136.3	1386.5	944.7	126.9	1005.8
*N*_50_	1106	351	595	921	249	577

fpc, fingerprinted contigs.

a Small contigs (average bacterial artificial chromosomes, BACs/contig = 2.6 for 6AS and 2.8 for 6AL) exclusively made by ltc. The average tag per BAC for the underlying clones was only 15 tags, equal to the value observed for singletons.

b The average number of tags per BAC for the underlying clones was 34 tags.

### BAC WGP™ tags facilitated *in silico* genetic anchoring of the 6A physical map

To facilitate efficient *in silico* anchoring, the short WGP™ tags underlying 6A physical contigs were extended by connecting them to the available wheat sequence information in three ways (Figures S1 and S2; Appendix S6). Firstly, WGP™ tags were connected to the available shotgun sequence contigs (obtained from IWGSC) of the bread wheat 6A chromosome arms (Appendix S6). This resulted in assigning 165–Mb of sequences (out of 433.6* *Mb) from WCS contigs to the wheat 6A physical map. Secondly, the previously assigned WCS contigs were connected to the progenitor genome sequences of *Triticum urartu* (an A–genome progenitor; Ling *et al.*, 2013), which resulted in assignments of 375* *Mb of sequences along the physical map. Thirdly, in order to enrich the physical map with further sequence information, an additional layer of sequences was added to sequences already assigned to 6A WCS and *T. urartu* using *Aegilops tauschii* (a D–genome progenitor; Jia *et al.*, 2013; Figures S1 and S2; Appendix S6). This provided a total of 157* *Mb of sequences from *Ae. tauschii* assigned to the 6A physical map. Considering the more distant relation of *Ae. tauschii* to bread wheat chromosome 6A, the effect of the *Ae. tauschii* sequence inclusion to our anchoring analysis was checked. We found that the incorporation of this sequence data set had a limited contribution to the overall anchoring of the physical map, with no negative effect on the accuracy of the genetic anchoring (Appendix S7). Altogether, the average cumulative sequence information per physical contig increased from 2144 nt using WGP™ tags to 11* *067 nt, simply by adding the available wheat sequence resources (Figure S2; Appendix S8). This number of sequences directly assigned to the physical map enabled sequence homology searches against all available genetic markers with known sequences, and thus provided the basis for the integration of genetic and physical maps *in silico* (Appendix S6). Genetic anchoring was performed for both ltc and fpc physical map assemblies; however, only the result of genetic anchoring performed for ltc assembly is shown here. This assembly was selected as the final 6A assembly because we observed that ltc provided a more robust assembly than fpc (see the following section).

**Figure 1:**
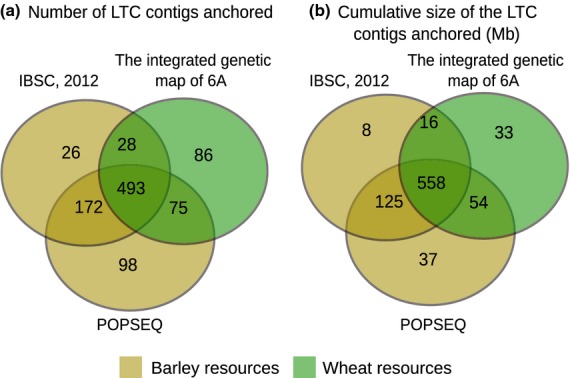
Contribution of different genetic map resources to the *in silico*-based anchoring of the wheat chromosome 6A physical map. (a) The number of ltc contigs that were anchored using one or a combination of different map resources: e.g. 493 ltc contigs were anchored by all three resources. (b) The corresponding cumulative size of the ltc contigs that were anchored using one or a combination of different map resources: e.g. 558 Mb was anchored by all three resources. The integrated genetic map of 6A (constructed in the current study; Appendix S6) refers to the combined genetic map derived from two highly dense wheat genetic maps, as previously described (Poland *et al.*, 2012; Cavanagh *et al.*, 2013). IBSC (2012) and POPSEQ (Mascher *et al.*, 2013) refer to the publicly available *Horedum vulgare* (barley) resources, including data sets from the International Barley Genome Sequencing Consortium (IBSC, 2012) and population sequencing (Mascher *et al.*, 2013), respectively.

First, ltc contigs were anchored to two highly dense wheat genetic maps (Poland *et al.*, 2012; Cavanagh *et al.*, 2013), which allowed us to genetically anchor 298 6AS and 384 6AL ltc-derived contigs. This genetically anchored a total of 661* *Mb out of the 1048* *Mb, which represents the cumulative contig map length (Figure 1; Appendix S9). By considering only genetically anchored ltc-built contigs, we were able to genetically anchor 132* *Mb of WCS contigs, 303* *Mb of sequence data from *T. urartu* and 129* *Mb from *Ae. tauschii* to the 6A genetic maps. The remaining unanchored 6A physical contigs were subjected to a second round of anchoring using the publicly available barley genomic resource. This was performed to provide researchers with an additional layer of anchoring information for the respective physical map. The barley-based anchoring was kept independent (Appendix S10) of the wheat-based anchoring. Therefore, in our analysis, we used 15* *719 high-confidence barley genes from the barley genome (IBSC, 2012), together with 723* *499 anchored barley WCS contigs from barley POPSEQ data (Mascher *et al.*, 2013). Using high-confidence barley genes we were only able to anchor 26 additional 6A physical contigs (8* *Mb), whereas 98 physical contigs from 6A (37 Mb) were exclusively anchored via barley population sequencing (POPSEQ) data (Appendix S10). Overall, 831 Mb (i.e. 79% of 6A physical contigs) were genetically anchored (Figure 1).

**Figure 2:**
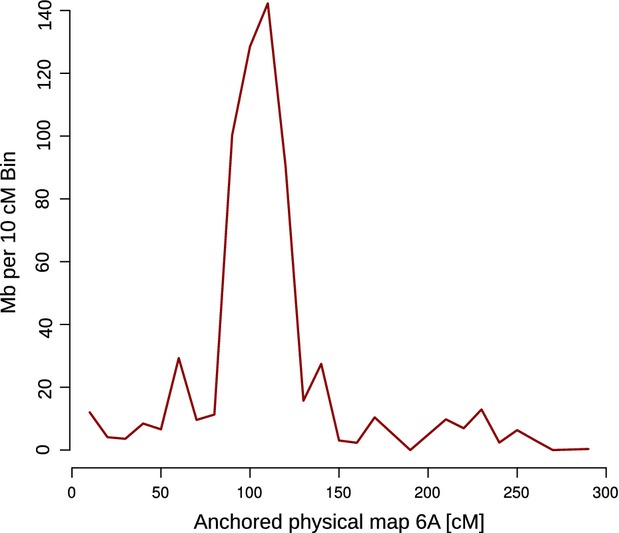
Estimated recombination frequency along wheat chromosome 6A. The underlying genetic map used for 6A genetic anchoring was divided into 26 bins, each of 10 cM in size. The physical map anchored to each bin measured the recombination pattern (Mb/cM) along the 6A chromosome. The genetic map is derived from integrating two wheat genetic maps (Appendix S6) developed previously (Poland *et al.*, 2012; Cavanagh *et al.*, 2013).

The large portion of the physical map anchored to the respective wheat genetic maps allowed for the analysis of recombination frequencies along the entire wheat 6A chromosome. For this purpose, an integrated wheat genetic map was constructed from the two aforementioned wheat genetic maps (Appendix S6). We then calculated the physical distance per 10–cM bins (Appendix S11). As expected, for plants with large genomes [i.e. *Zea mays* (maize; Anderson *et al.*, 2003), *Hordeum vulgare* (barley; Kunzel *et al.*, 2000) and wheat (Lukaszewski and Curtis, 1993)], we were able to reconfirm that recombination greatly increased from the centromere towards the telomeres (Figure 2). These detailed estimates related to recombination frequencies along 6A will be most useful for future map-based cloning and gene identification projects.

### Comparison between ltc and fpc assembly at different cut-off values

**Figure 3:**
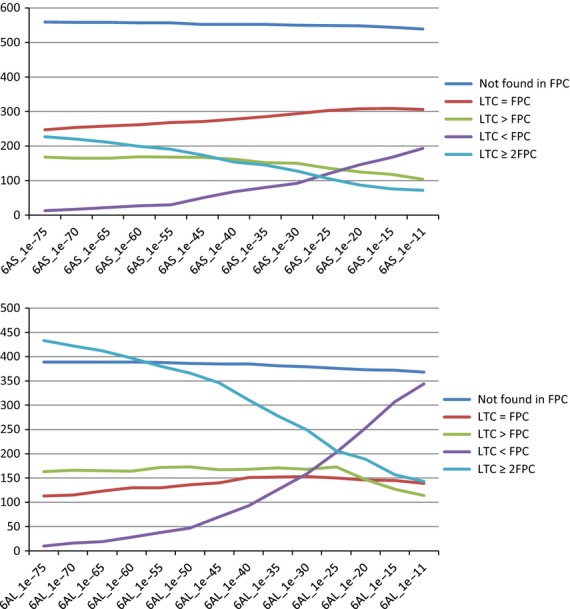
Comparison of linear topological contig (ltc)-derived physical contigs with fingerprinted contigs (fpc) at different stringencies. Different classes of ltc-derived contigs were identified when compared with fpc assemblies that include the first class, which are contigs that were exclusively made by ltc (not found in fpc). The number of such ltc-specific contigs was relatively constant for both of the 6A arms when compared with fpc assemblies at various stringencies. The second class contained contigs that were identical between assemblies made by both tools (ltc = fpc, i.e. BAC composition and order were the same in corresponding ltc and fpc contigs). This class showed a slight increase when the fpc stringency was lowered. The third class includes ltc > fpc (i.e. ltc contigs longer than fpc contigs), which refers to contigs with the same backbone, whereas more BACs were added to the end of corresponding contigs via ltc. This class also showed a slight decrease in number by lowering the stringency in fpc. The two remaining classes include ltc < fpc (i.e. ltc contigs that are shorter than fpc contigs) and ltc ≥ 2fpc (i.e. ltc-made contigs, the BACs of which were assembled into two or more different contigs via fpc). Both classes showed higher differences in number across the fpc assemblies that are mainly explainable by end-to-end merging during fpc assembly. For example, a decrease in the number of ltc ≥ 2fpc from 1e^−30^ to 1e^−25^ is likely to have resulted from the merging of the two corresponding fpc contigs at 1e^−25^. Therefore, contigs of 1ltc = 2fpc at a higher level of fpc stringency later became 1ltc < 1fpc (or 1ltc = 1fpc) at a lower level of stringency. In both chromosome arms, identical classes show a relatively similar trend of variation across fpc assemblies, although they may contain different numbers of contigs in each of the arms.

**Figure 4:**
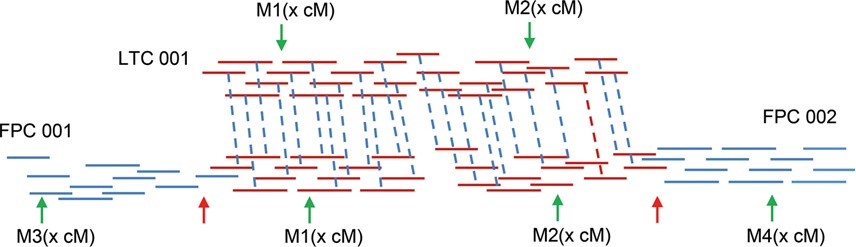
Schematic representation of a case of conflict (ltc ≥ 2fpc) in fingerprinted contigs (fpc) versus linear topological contigs (ltc), as a reference. Anchored genetic markers (M) were used to confirm the corresponding bacterial artificial chromosome (BAC) contig structure. Three situations were observed. Status 1: concordant M_1_ and M_2_ genetic positions, with the genetic position for the corresponding marker in at least one fpc contradictory; ltc 001 correctly assembled. Status 2: non-concordant M_1_ and M_2_ genetic positions, with the genetic position of the corresponding marker in both fpc contigs reconciled; ltc 001 not correctly assembled. Status 3: concordant M_1_ and M_2_ genetic positions, with the genetic position for the corresponding marker in both fpc contigs reconciled; all contigs correctly assembled. Red/blue bars, BAC clones underlying BAC contigs; dashed bars, connect shared BACs; green arrowheads, genetic marker position; red arrowheads, region of low BAC coverage.

To date, physical maps of four arms of wheat chromosomes, including 1AS, 1AL, 1BS, 1BL and 3B, have been reported (Paux *et al.*, 2008; Breen *et al.*, 2013; Lucas *et al.*, 2013; Philippe *et al.*, 2013; Raats *et al.*, 2013). With the exception of chromosome 3B, both ltc and fpc tools have been used to assemble wheat chromosome physical maps. A comparison was performed between the two tools for wheat chromosome arm 1AL and showed that the ltc assembly provided significantly higher accuracy (Breen *et al.*, 2013); however, the previous study only described the advantages of ltc by comparing the ltc assembly with a single fpc assembly constructed at 1e^−45^ and by using HICF data (and not WGP™) as the input (Breen *et al.*, 2013). In our study, we compared contigs obtained by ltc as the reference assembly with individual fpc assemblies (Table S1) generated at different stringencies to assess the differences between the ltc and the fpc assemblies employing WGP™. This allowed for the identification of the most robust physical map for 6A. Our comparison distributed an assortment of ltc-made contigs into five groups (Figure 3; Tables S2 and S3; Appendix S12). The ltc ≥ 2fpc group (ltc-derived contigs for which BACs were assembled into two or more different contigs via fpc; cases of conflict; Figure 4) was considered the most suitable group for comparing the two assembly platforms.

The number of such ltc-built contigs (ltc ≥ 2fpc) was found to decrease with declining fpc assembly cut-off values. This was explained by end-to-end merging during fpc assembly. To check whether contig merges were accurately formed via fpc while decreasing assembly cut-off, all ltc* *≥* *2fpc cases were visualized and inspected (Figure 4). This inspection was only performed for cases of conflict between ltc and fpc assemblies at a 1e^−11^ fpc cut-off for each chromosome arm (Figure 4; Appendices S13 and S14). This cut-off was selected because it was closest to the ltc cut-off value (1e^−10^), and thus was considered to represent the most comparable cut-off value. In the case of 6AS, 72 conflicts were found for which a particular set of overlapping BACs were always shared between ltc and corresponding fpc counterparts. These BACs were also similarly ordered along the respective contigs (Figure 4, red-colored BACs; Table 4).

**Table 4:** Cases of conflict (ltc ≥ 2fpc) for which marker data allowed further confirmation of contig structure

Chromosome arm	6AS	6AL
No. of conflicts	72	143
No. of conflicts for which informative markers were available^a^	22	26
Engaged fpc contig(s) is chimeric	17	20
Engaged ltc contig is chimeric	4	4
The ltc contig corresponds to the ends of the respective fpc contigs^b^	1	2

fpc, fingerprinted contigs; ltc, linear topological contigs.

a Genetic markers from the same map were anchored to different parts of the same contig.

b Neither the ltc contig nor the respective fpc contigs could be flagged as chimerical on the basis of anchored genetic markers.

We then analyzed whether the conflict could be resolved by consulting genetic markers anchored to corresponding BAC clones. For 22 of the 72 6AS cases of conflict, the corresponding affected ltc- and fpc-made contigs were provided with a sufficient number of anchored genetic markers (informative markers). Such markers allowed us to ascertain whether the different parts of affected contigs had been correctly overlapped/merged and, if so, whether the conflict could be resolved. In the majority of cases (17 out of 22), genetic markers revealed a false assembly of different fpc-derived contig parts (e.g. a chimerical fpc contig was built; Figure 4, status 1). For the remaining five cases, either the ltc-built contigs were chimeric (four cases; Figure 4, status 2) or the respective BACs were correctly assembled by both fpc and ltc tools (one case), and thus corresponding conflicts were resolved (Figure 4, status 3). We achieved a similar result for 143 conflicts identified for 6AL (Table 4). Therefore, we concluded that the ability of fpc to form longer and consequently lower numbers of contigs, while decreasing the assembly cut-off value, could potentially produce higher numbers of chimerical contigs as a result of false BAC contig end-to-end merges.

We also observed a highly consistent number of ltc-specific contigs (small contigs, average BAC/contig* *=* *2.6–2.9) compared with fpc assemblies. These ltc-derived contigs (539 for 6AS and 368 for 6AL, compared with fpc at 1e^−11^) were composed of BACs that were left out as singletons in the initial fpc build at 1e^−75^ (Table S1), where BACs were assembled into contigs only if they shared >70% of WGP™ tags (Paux *et al.*, 2008). BAC clones containing fewer tags (an average of 15 tags/BAC, compared with 34 tags/BAC for the rest of the assembly) were incorporated into contigs via ltc because the initial assembly was performed at a lower stringency (1e^−2^ cut-off). Therefore, such ltc-specific small contigs resulted in a higher number of ltc-made contigs, and consequently had higher chromosomal arm coverage, as they most likely cover the same regions as larger contigs. Although ltc had an artificially generated larger number of small contigs, the robustness of BAC order and overlap identification for the remaining contigs was significantly higher in ltc versus fpc assemblies (see cases of conflict above). For this reason, we considered the entire ltc-based assembly to be a more reliable physical map for subsequent analyses, including synteny analysis, MTP selection and future BAC-based sequencing of wheat chromosome 6A.

### Efficacy of fpc at improving ltc-made assembly

To test whether gaps between ltc-derived contigs could be closed via fpc-assembled BACs, ltc-derived contigs were aligned against fpc assemblies using a 1e^−50^ cut-off as a reference. This was in contrast to the aforementioned comparisons, where ltc was used as a reference. We selected this stringently formed FPC assembly to ensure the robustness of overlap among BACs of the corresponding contigs. Using this approach, we identified 25 fpc-built contigs for 6AS and 45 fpc-built contigs for 6AL in which BAC clones were represented in more than two ltc-made contigs (Table 5). These ltc-derived contigs were thus considered potentially mergable or potential scaffolds because the gap between two ltc-made contigs could be bridged using a robustly formed fpc-built contig. All of these scaffolds were depicted as individual images according to BAC position to enable a visual inspection of their structure (Figure S3). Only scaffolds for which the corresponding fpc-made contig was composed of BACs from the ends of two different ltc-made contigs were flagged as potentially true scaffolds (10 for 6AS; 17 for 6AL); however, those fpc-derived contigs that, for example, contained BACs from the end of one ltc-made contig and BACs from the middle of another were rejected (almost 60% for each arm; Figure S3). These rejected scaffolds most likely represent falsely assembled BACs via fpc (chimerical contigs).

**Table 5:** Scaffolding linear topological contigs (ltc) using fingerprinted contigs (fpc) assembled at a cut-off of 1e^−50^, for which the same set of bacterial artificial chromosomes (BACs) was employed

Chromosome arm	6AL	6AS
No. of potential scaffolds	45	25
Scaffolding was structurally allowed^a^	17	10
Structurally allowed scaffolds for which genetic markers were available^b^	6	4
Structurally allowed scaffolds were supported by genetic markers	3	3
Structurally allowed scaffolds were not supported by genetic markers	3	1

a The corresponding fpc contig was only composed of BACs from the end of two different ltc contigs, and not from the end of one ltc and the middle of another (not allowed).

b The corresponding ltc contigs were provided by genetic anchoring information.

Using these scaffolds we then tested whether any anchoring information could support scaffold accuracy. For 6AS, genetic markers were available for four such scaffolds, of which one was confirmed genetically; for 6AL, we found three cases out of six (Table 5). This result indicated that the efficacy of fpc assemblies (at 1e^−50^) at improving ltc-derived assemblies was relatively low. In general, this might indicate that the complexity of the wheat sequence restricted the ability of fpc, using WGP™ data as input, to construct robust contigs, even at the initial higher stringency (in this case 1e^−50^).

### Scaffolding the ltc assembly using WGP™ tags and shotgun sequence contigs

Publicly available *T. urartu* sequence contigs (Tu contigs) were used to determine whether they allow for the bridging of ltc-made contigs and building of ltc scaffolds. We checked whether a single Tu contig exclusively matched terminal parts of two different ltc-derived contigs (Appendix S15). Using Tu contigs as a proxy, we identified 84 6AS and 65 6AL potential scaffolds (Table 6; Appendix S16). Those ltc-made contigs engaged in scaffolding were relatively small (average BAC/contig* *=* *5 6AS and 17 6AL), and therefore could not have a significantly positive effect on the L_50_ of the overall BAC assembly; however, we asked whether ltc-derived contigs involved in such scaffolds were supported by genetically anchored markers to validate our approach (with a similar approach as described in Figure 4). For 6AS, we found only four of these scaffolds, of which two could be genetically validated; for 6AL, 12 scaffolds with genetic markers were provided, of which eight were genetically confirmed (Table 6). Although genetic markers were not available for structural confirmation of all the scaffolds constructed, we kept and reported all formed scaffolds (Appendix S16) because they could potentially support and/or guide future BAC-based sequencing and sequence assembly of the respective physical contigs.

**Table 6:** Scaffolding linear topological contigs (ltc) using the underlying whole-genome profiling (WGP™) tag and whole-genome shotgun sequence contigs of *Triticum urartu*^a^

Chromosome arm	6AL	6AS
No. of potential scaffolds^b^	65	84
No. of scaffolds for which genetic markers were available^c^	12	4
No. of genetically confirmed scaffolds	8	2
No. of not genetically confirmed scaffolds	4	2

a This investigation was performed using only a subset of *T. urartu* sequence info (*T. urartu* scaffolds): 90.7 Mb, with an L_50_ of 64 532 nt.

b Scaffolding was allowed if the corresponding sequence contigs hit at least three WGP™ from the ends of only two ltc contigs.

c The corresponding ltc contigs were provided by genetic anchoring information.

### ‘Gene decoration’ of the newly formed anchored physical map of wheat chromosome 6A

**Figure 5:**
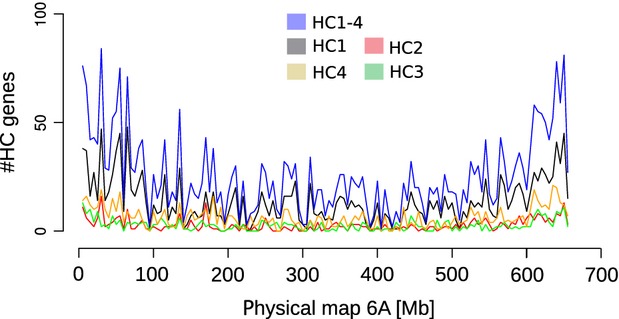
Estimated gene distribution along wheat chromosome 6A. Chromosome 6A gene-annotated whole chromosome survey sequencing contigs were linked to whole-genome profiling tags underlying bacterial artificial chromosome (BAC) contigs. High-confidence (HC) genes represent annotated genes, wherein HC1 are genes with >70% coding sequence–reference gene overlap in *Brachypodium distachyon*, *Oryza sativa* (rice) or *Sorghum bicolor*, whereas the other HC2–HC4 classes have a smaller overlap (IWGSC, In press).

Chromosome 6A shotgun sequences (6A WCS contigs) have already been annotated for genes (IWGSC, In press), which established 5024 genes grouped into four confidence classes (HC1–HC4). Of these, 2531 genes were assigned to the HC1 category because ≥70% of coding sequences overlapped with a reference gene in *B. distachyon*, rice or *S. bicolor*. The remaining confidence classes (HC2–HC4) had less overlap with reference genes (IWGSC, In press); however, in our analysis of the total 5024 HC1–HC4 genes, 3359 genes (1667 6AS; 1692 6AL) were assigned to the anchored portion of the 6A physical map (i.e. genetically positioned genes). This was accomplished by assigning the corresponding WCS contigs to the WGP™ tags (Appendix S6). We then calculated the gene density on this newly formed physical map along 6A by dividing the chromosome length in bins of 5* *Mb (Figures5 and S4). In more telomeric bins we found a maximum of 84 genes, whereas in the centromeric region with low recombination the number of genes decreased dramatically to less than 20 (Figure 5). Moreover, this analysis revealed a general correlation between the recombination rate pattern and distribution of genes along the chromosome (Figures2 and 5).

### Synteny-based approach addressed 6A evolutionary relations with model grass genomes

To analyse the completeness of the anchored physical map and to gain insight into the evolutionary origin of chromosome 6A, physical contig-associated sequences were compared against reference genomes (Appendix S6). Associated sequence information, including WGP™ tags, WCS, *T. urartu* and *Ae. tauschii* contigs, were compared with coding sequences from other grass genomes of *Hordeum vulgare* (Hv), *Brachypodium distachyon* (Bd), *Oryza sativa* (Os), and *Sorghum bicolor* (Sb). Anchored sequences showed sequence homology to 2799 Hv, 2455 Bd, 2539 Os and 2465 Sb genes. Of these genes, 40.0–48.5% matched syntenic chromosomes of the three genomes, including Bd3, Os2 and Sb4. These results are comparable with those reported previously (40.2–59.7%) for syntenic genes of 6B (Tanaka *et al.*, 2014).

**Figure 6:**
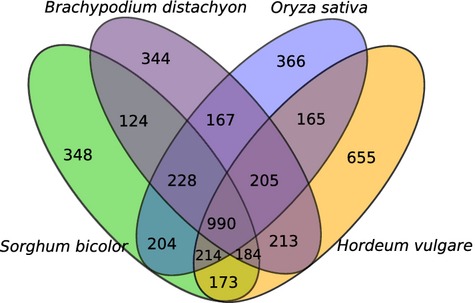
Venn diagram of wheat chromosome 6A physical map-associated gene distribution with significant similarity to *Brachypodium distachyon*, *Oryza stiva* (rice) and *Sorghum bicolor*. Chromosome 6A physical map-associated sequence information, including whole chromosome survey sequencing, *Triticum urartu* and *Aegilops tauschii* contigs, were compared with coding sequences from model grass genomes. In *Hordeum vulgare*, all genes including genetically anchored and unanchored genes were considered (IBSC, 2012).

**Figure 7:**
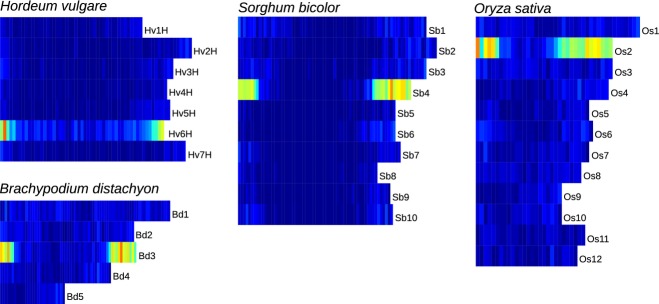
Wheat chromosome 6A physical map-associated genes mapped to barley (*Hordeum vulgare*; Hv), *Brachypodium distachyon* (Bd), rice (*Oryza sativa*; Os) and *Sorghum bicolor* (Sb) genomes. In barley, only genetically anchored genes are depicted (IBSC, 2012). Gene overlaps ranged from 0 (dark blue) to the maximum, shown in dark red.

Figure 6 depicts the 6A gene distribution among model grass genomes. We observed that the number of wheat coding sequences shared with at least three species (1994 genes) was higher than the genes presented in two (873 genes) or a single species (1713 genes). Proteins with significant similarity (seed length of 20, identity ≥75%; http://www.vmatch.de) to wheat sequence information were plotted along the chromosomes (Figure 7). The average syntenic gene content was calculated as the number of matching wheat proteins in a window of 1* *Mb, without overlap. In this analysis, 6A sequence information showed homology with genes/proteins located on chromosome 6H of Hv (Hv6H), Bd3, Os2 and Sb4, but not with any other chromosomes of these genomes (Figure 7), which is consistent with previous reports (Salse *et al.*, 2008; TIBI, 2010; Brenchley *et al.*, 2012; IBSC, 2012).

## Discussion

The current study is the German contribution to the IWGSC. This international consortium has aimed to sequence wheat chromosomes and/or chromosome arms individually, with the construction of the respective BAC-based physical maps as a necessary intermediate step. In this context, we report here the successful application of WGP™ together with the contig assembly tool ltc to robustly assemble BAC clones into 1113 contigs for the long arm and 1217 contigs for the short arm, representing the wheat chromosome 6A physical map. To date, physical maps of four arms of wheat chromosomes, including 1AS, 1AL, 1BS and 1BL, as well as the entire chromosome of 3B, have been reported (Paux *et al.*, 2008; Breen *et al.*, 2013; Lucas *et al.*, 2013; Philippe *et al.*, 2013; Raats *et al.*, 2013). The 6AS chromosome arm (336* *Mb) has the closest estimated size to that of 1BS (314* *Mb; Šafář *et al*., 2010), for which an initial assembly (before manual end-to-end merging) of 254 ltc-derived contigs (with six or more clones) was reported. Excluding small contigs (with five or fewer clones) from our 6A assemblies would result in 293 (368* *Mb) and 545 (459* *Mb) contigs for the short and long arms, respectively. Therefore, the number of contigs obtained for 6AS is comparable with the total number of contigs in the initial assembly of 1BS; however, in contrast to the final assembly (after manual end-to-end merging) of 1BS that resulted in 57 scaffolds, no further contig merging or scaffold construction was considered in the final 6A assemblies, as this must be guided by highly reliable and robust genetic maps. Otherwise, the complexity and repeat content of the wheat genome could potentially hamper any manual contig merging or super-contig construction. Excluding small contigs, we obtained more than 100% coverage for 6AS (109%) and 6AL (124%) assemblies. This is most likely because the original chromosomal arm sizes have been underestimated.

Overall, we were able to anchor 79% of the physical contigs into corresponding genetic maps, which is greater than the aforementioned wheat chromosomal physical maps, including 1BL (74%), 1AS (74%), 1AL (∼75%) and the first version of 3B with a 56% anchored physical map. Nevertheless, the 1BS physical map contained 83% of the contigs anchored to the respective genetic maps because of the low number of contigs (Raats *et al.*, 2013). The availability of extended WGP™ tags allowed for the direct placement of 3843 genes into a physical map, which was comparable with the recent physical map for barley, where an average of ∼3700 genes could be assigned to each chromosome (IBSC, 2012). This high level of genetically anchored physical maps and their respective genes provides a more efficient way to clone agronomically important genes/QTLs located on 6A. Fine-mapping and the identification of genes underlying these important QTLs have been inhibited in wheat, mainly by technical constraints linked to its genome size (17* *Gb), repeat content (>80%) and genomic redundancy (presence of three highly homologous genomes: A, B and D). These limitations may explain why very few wheat genes have been cloned (Krattinger *et al.*, 2009). Therefore, chromosomal BAC-based physical maps are of utmost importance to promote and simplify positional cloning in this large genome.

**Figure 8:**
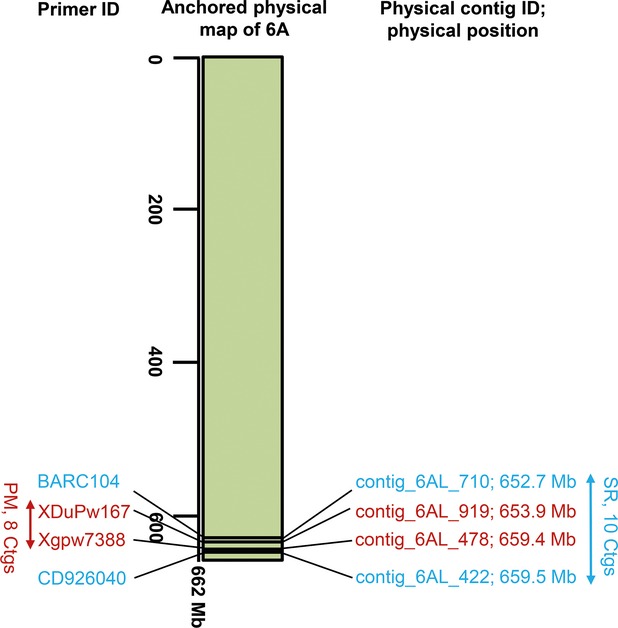
Physical map intervals containing agronomically important genes on wheat chromosome 6A. Primers or corresponding PCR-amplified sequences were used to define the corresponding interval. An *in silico* sequence homology search was performed to connect primer sequences or corresponding PCR-amplified sequences to sequence information underlying the 6A physical map; PM, powdery mildew-resistant gene (Muranty *et al.*, 2009); SR, stem rust resistance (Dubcovsky *et al.*, 2011; Simons *et al.*, 2011). Bi-directional arrows indicate gene containing intervals; corresponding genetic and physical information of each interval have been highlighted with the same color.

In this study, the comprehensive and integrated 6A physical map localized some genetic determinants to the corresponding physical map, and provided information required for the development of tightly linked genetic markers (Figure 8). Such loci include one resistance QTL that is important against the wheat disease *Fusarium* head blight (Schmolke *et al.*, 2005; Holzapfel *et al.*, 2008), the stem rust resistance gene *Sr13* (Dubcovsky *et al.*, 2011; Simons *et al.*, 2011), an anti-xenosis gene against a new aphid biotype (Castro *et al.*, 2005) and QTLs involved in adult plant resistance to powdery mildew (Muranty *et al.*, 2009), as well as greater seedling vigor (Spielmeyer *et al.*, 2007). All of these QTLs/genes had already been genetically mapped to 6A; however, in the current study, only two corresponding gene intervals were successfully localized to the 6A physical map. For the remaining genes, either the corresponding primer/marker sequence information was not publicly available or the marker sequence could not be detected in our 6A-connected sequence data set. For the identified intervals, the respective information, including the number of genes and physical contigs assigned to a respective region, were identified (Figure 8). This analysis shows the usefulness of our physical map and represents an unprecedented opportunity to accelerate detailed gene studies, including positional cloning, and ultimately wheat breeding programs.

In the absence of the aforementioned wheat physical maps, comparative genomics and collinearity between wheat and related grass genomes have been the method of choice for map-based cloning in wheat. Previous strategies have been shown to be very arduous, costly and to require a variety of genomic resources. For example, initial attempts at cloning the wheat *Lr34/Yr18* locus failed because of a lack of sufficient collinearity between wheat and the small rice and *B. distachyon* genomes (Spielmeyer *et al.*, 2008). Although conserved synteny was of great support in narrowing down respective gene-containing intervals, the region carrying *Lr34/Yr18* is absent in both rice and *B. distachyon* syntenic segments, validating the hypothesis that grass resistance genes have less conserved micro-colinearity (Leister *et al.*, 1998). Access to robustly assembled hexaploid wheat genome physical maps is the key to expediting future wheat genome sequencing efforts.

Together with the 6A physical map, we were also able to estimate the rate of recombination along 6A. A general agreement between the pattern of recombination rate and gene distribution along chromosomes was observed, similar to an earlier report (Erayman *et al.*, 2004). This report revealed a correlation between recombination gradient and gene distribution by physically mapping 3025 genes/QTLs to 334 deletion break points that spanned all seven wheat chromosomal groups. This eventually enabled the co-localization of gene- and recombination-rich regions along wheat chromosomes. In our study, the highest density of genes was found for more distal regions of 6A and where recombination rates reached their maximum. In the centromeric area of 6A, however, where recombination rates were the lowest, the number of genes per physical unit also declined dramatically. Such suppression of recombination limits our genetic anchoring resolution for the (peri)centromeric area in which a high number of physical contigs are anchored with unclear order. Therefore, different mapping approaches (e.g. radiation hybrid mapping that is independent of recombination; Kalavacharla *et al.*, 2006) should be used in the future to precisely order contigs in this region. In any case, the recombination estimates along 6A provided are imperative for accomplishing a more efficient isolation of important genes/QTLs via map-based cloning, as genes located in regions with high recombination rates are more accessible to map-based isolation (Jander *et al.*, 2002).

### The efficiency of WGP™ technology and the advantage of the ltc assembly tool over fpc

This study reports the application of WGP™ on individual wheat chromosome arms and effectively confirms how well WGP™ technology works on arms with highly repetitive sequences (>80%) to accurately form a physical map. The advantage of WGP™ over conventional HICF-BAC fingerprinting assembly in wheat has previously been discussed (van Oeveren *et al.*, 2011; Philippe *et al.*, 2012). In this respect, the robustness and quality of a BAC assembly may be justified with the quality and quantity of information (e.g. tag length and density in WGP™ or band size and similarity in HICF) provided for pairwise comparison of BACs and the subsequent establishment of respective contigs. In our study, both tag length and density were significantly improved by 64% compared with the values obtained in the WGP™-based pilot study performed on wheat chromosome 3B (Philippe *et al.*, 2012). Our improved WGP™ results were achieved by applying a different combination of restriction enzymes with higher cut-site frequency (*Hin*dIII/*Mse*I), and by increasing the initial sequence read length to 100 nt, as previously suggested (Philippe *et al.*, 2012). Therefore, by using additional sequence information per BAC, together with higher stringency (tolerance value = 0) during assembly and a more efficient contig formation tool (ltc), we propose that a highly accurate BAC-based physical map of 6A has been developed.

The superior performance of ltc using HICF data compared with fpc was recently illustrated in wheat (Breen *et al.*, 2013; Raats *et al.*, 2013). Here, similar conclusions were derived while applying WGP™ tags for BAC assembly of both 6A arms. ltc-derived contigs could be classified into five groups when compared with fpc-made contigs obtained at a given cut-off value. The striking difference between ltc and fpc is reflected in ltc-built contigs (i.e. LTC ≥ 2fpc; cases of conflicts) in which underlying BAC clones were assembled in more than one contig when compared with fpc assemblies. In a randomly selected sample (ltc ≥ 2fpc, fpc at 1e^−11^), genetic anchoring revealed that in 71% of such cases, the corresponding fpc-derived contigs were incorrectly merged, whereas for ltc, this was only 13%. Moreover, by visually inspecting mis-assembled fpc-made contigs, we often observed that inconsistent cases had highly degenerate BAC coverage compared with other contig parts (Figure 4, red arrowheads). Low-coverage regions were rarely detectable in corresponding ltc-derived contigs for which multiple fpc contigs were available. Low-coverage regions in fpc-built contigs are most likely the result of false end-merging during the stepwise reduction of assembly stringency in fpc.

Our observations further demonstrate the advantages of the ltc-based BAC assembly for large genomes containing large numbers of repetitive elements. This is in agreement with a previous report that showed ltc was more efficient at forming BAC contigs using HICF (Breen *et al.*, 2013; Raats *et al.*, 2013). In addition, we showed that by applying a more reliable technology (i.e. WGP™), fpc performance is still considerably lower than that of ltc in physical map assembly. Therefore, we highly recommend combining WGP™ fingerprint methodology together with ltc assembly software for future physical mapping efforts in wheat.

## Experimental Procedures

### Chromosome sorting and construction of BAC libraries

The 6AS arm was flow-sorted from a double ditelosomic line of wheat carrying both arms of chromosome 6A as telosomes (2*n* = 40 + 2t6AS + 2t6AL), whereas the 6AL arm was purified from a ditelosomic line carrying only the 6AL arm as a telocentric chromosome (2*n* = 40 + 2t6AL), according to Vrana *et al*. (2000). Both flow-sorted telosomes were derived from cv. Chinese Spring. The identity and purity in the sorted fractions was checked by fluorescence *in situ* hybridization using probes for the telomeric repeat and for the GAA repeat (Janda *et al.*, 2006). Chromosome arm-specific BAC libraries were constructed as described by Šimková *et al*. (2011). In order to estimate the average insert size, 160 BAC clones were randomly selected from each of the libraries and analyzed as described in Janda *et al*. (2006).

### WGP™ data production

A 3D format of BAC pools was made for each BAC library (Appendix S17). High-concentration BAC DNA was subsequently isolated from pooled BACs, followed by WGP™ sample preparation, as previously described (van Oeveren *et al.*, 2011). Briefly, restriction ligation templates were prepared from pooled BAC DNA by digestion using *Hin*dIII and *Mse*I, followed by ligation of adaptor sequences containing sample identification tags (barcodes), PCR amplification and the pooling of respective PCR products. Sequencing of the resulting amplified cluster was performed using the Illumina HiSeq with a 100–nt read length. Sequence reads were used for WGP™ tag production (see Appendix S1), which included barcode and restriction site identification, deconvolution of reads as WGP™ tags to the individual BACs and the filtering of WGP™ tags using various quality controls. This filtering pipeline was used to eliminate tags matching vectors, *E. coli* or chloroplast sequences. Tags containing homopolymer sequences ≥5 nt were considered uninformative (i.e. with a high chance of being present in more than one BAC in a particular plate). Moreover, tags potentially introducing ambiguities were also eliminated (i.e. those present in >12 BACs).

### BAC contig assembly

To operate the BAC assembly, we used ltc (Frenkel *et al.*, 2010) and fpc 9.4 (http://www.agcol.arizona.edu/software/fpc/). Both employ the same metric or so-called Sulston score. In the case of ltc, as the tolerance could be set at the best stringency (tolerance* *=* *0), the initial net of significant clone overlaps obtained at a 1e^−2^ cut-off was considered. Corresponding subnets were then obtained at a 1e^−10^ cut-off and used for contig formation. All contigs with at least two clones were exported into fpc format and checked for linear topology. All contigs with a width ≥2 were checked and split manually to obtain linear contigs. If only one clone explained the nonlinearity, the contigs were left as such because this nonlinearity was likely to be caused by a lack of WGP™ tags in the corresponding clone (Philippe *et al.*, 2013). Further parameters required to establish the ltc physical map were as follows: a tolerance of 0; gel length of 111 000; N_bands_Sulston (number of bands for Sulston score calculation) equal to gel length; and a minimum contig size of two clones. Adaptive clustering was performed using the following criteria: a 1e^−3^ cut-off (while the initial value to make the net of significant clone overlaps was set to 1e^−2^) and a step size of 1, with seven steps. MTP clones were selected applying the aforementioned parameters in the ltc program. Because of the low number of genetic markers and the lack of adequate anchoring, no further contig merging or supercontig construction was performed.

In addition to ltc, contig assembly was also performed via fpc to compare the performance of each tool in physical map construction. Briefly, the initial fpc assembly was performed with a 1e^−75^ cut-off. This was subsequently run through single-to-end and end-to-end merging (Match, 1; From End, 13) at 13 sequentially higher cut-offs (thus, lower stringency) that ended up at 1e^−11^, as was suggested for a WGP™-based strategy in wheat (Appendix S4; Philippe *et al.*, 2012).

## Supporting Information

Figure S1 Steps for the elongation of WGP™ tags by connecting them to the available 6A related sequence information.

Figure S2 Different quantities of publicly available sequence information were connected with the physical contigs using the underlying WGP™ tags.

Figure S3 An example of homology between ltc and fpc contigs (as reference). ltc contigs were aligned against fpc contigs.

Figure S4 Different gene classes assigned to the physical contigs.

Table S1 Reduction in number of contigs and singleton assembled using fpc as a result of decreasing cut-off value.

Table S2 Comparison of 1214 ltc-assembled physical contigs of 6AS with fpc at different stringencies.

Table S3 Comparison of 1108 ltc-assembled physical contigs of 6AL with fpc at different stringencies.

Appendix S1 Whole-genome profiling (WGP™) of 6A chromosome arms.

Appendix S2 6AS ltc-derived physical map.

Appendix S3 6AL ltc-derived physical map.

Appendix S4 The fpc-based assembly of the WGP™-based BAC fingerprints.

Appendix S5 The fpc-based BAC assembly.

Appendix S6 Construction of an *in silico* anchored, gene-decorated physical map.

Appendix S7 The effect of incorporating *Ae. tauschii* sequences to the overall anchoring of the 6A physical map.

Appendix S8 6A ltc contigs and the associated sequence information.

Appendix S9 6A ltc-derived contigs genetically anchored to the genetic maps of wheat.

Appendix S10 6A ltc-derived contigs anchored to barley genomic resources.

Appendix S11 An estimation of the recombination frequencies along the chromosome.

Appendix S12 Complementary description of five different classes of ltc contigs when compared with individual assemblies obtained via fpc.

Appendix S13 6AS final fpc-derived physical map with a cut-off at 1e^−11^.

Appendix S14 6AL final fpc-derived physical map with a cut-off at 1e^−11^.

Appendix S15 Scaffolding the ltc contig.

Appendix S16 6A ltc-derived contigs mergable via Tu contigs (ltc scaffolds).

Appendix S17 BAC pooling scheme for WGP™ data production.
