# Supplementary figures and images for: Whole-genome profiling and shotgun sequencing delivers an anchored, gene-decorated, physical map assembly of bread wheat chromosome 6A
Source: Plant J. 2014 May 9;79(2):334–47. doi: 10.1111/tpj.12550 (PMC4241024; doi:10.1111/tpj.12550)

- 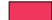 *Aegilops tauschii*
- 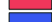 *Triticum urartu*
- 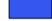 6A WCS contigs
- 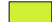 WGP tags
- 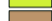 6A clones

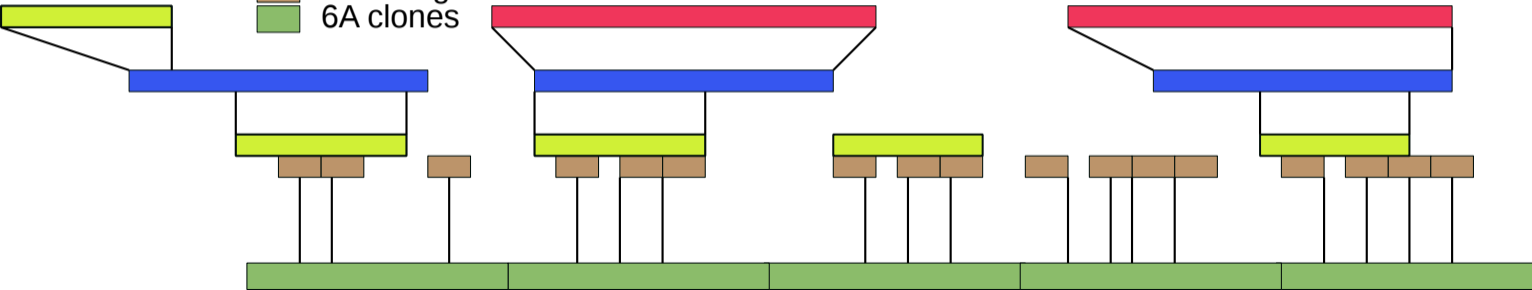

Supplement: Figure S1 — Steps for the elongation of WGP™ tags by connecting them to the available 6A related sequence information. [file tpj0079-0334-SD1.pdf]

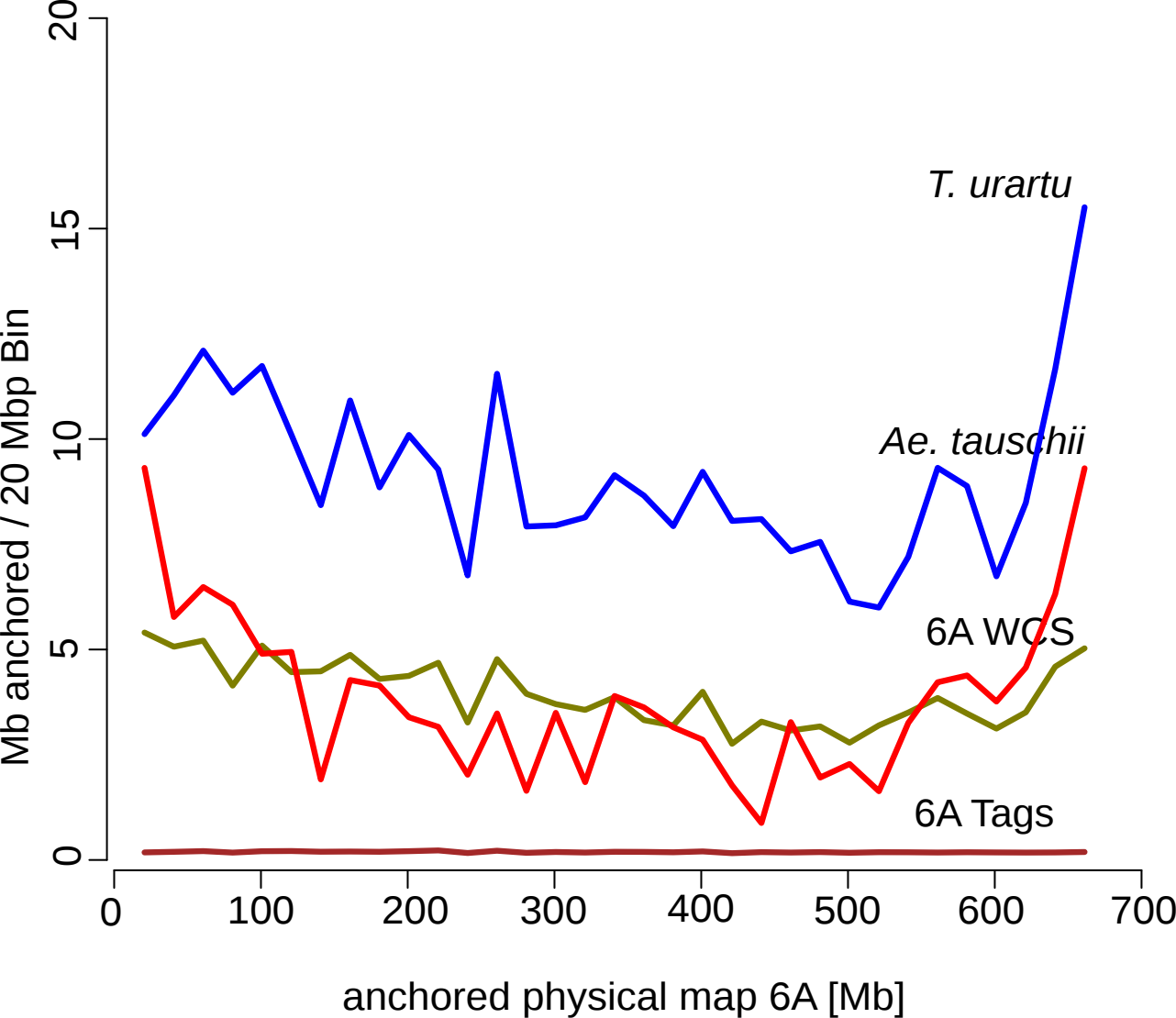

Supplement: Figure S3 — An example of homology between ltc and fpc contigs (as reference). ltc contigs were aligned against fpc contigs. [file tpj0079-0334-SD3.pdf]

FPC\_1069

LTC\_889

LTC\_1072

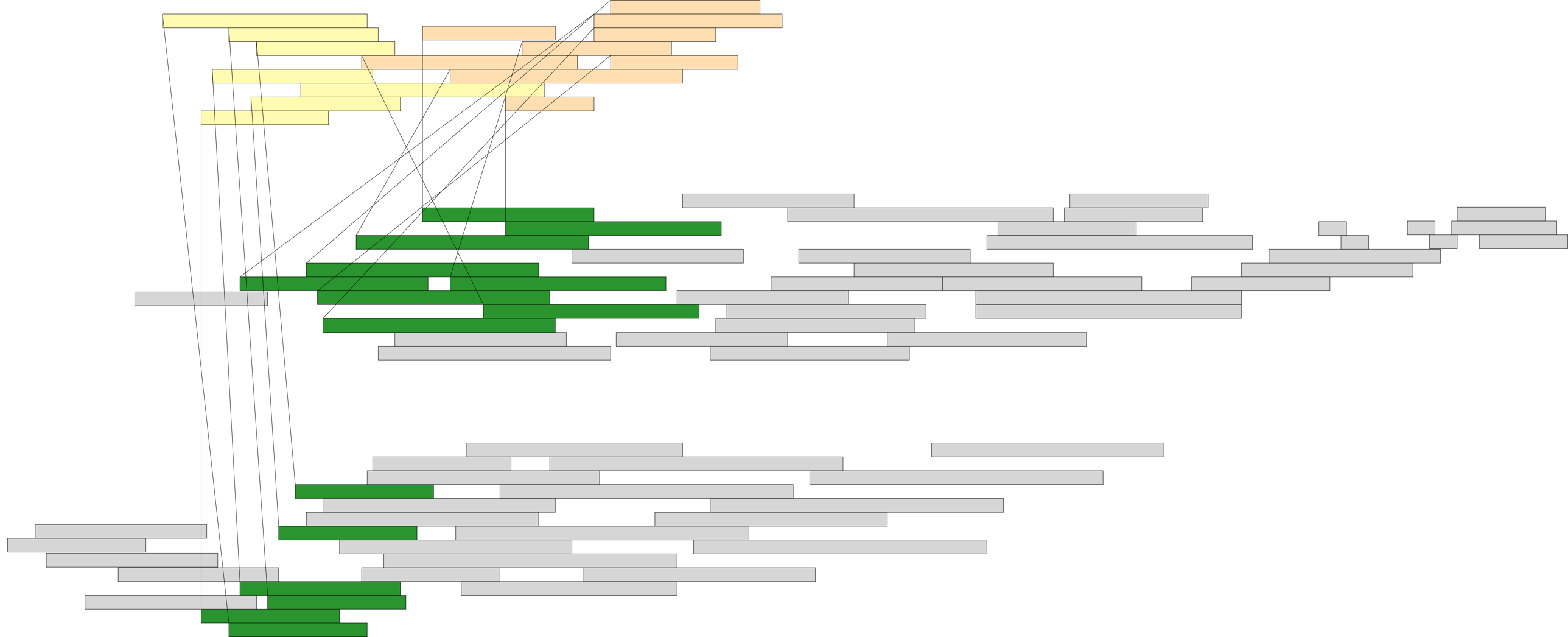

Supplement: Table S1 — Reduction in number of contigs and singleton assembled using fpc as a result of decreasing cut-off value. [file tpj0079-0334-SD5.pdf]

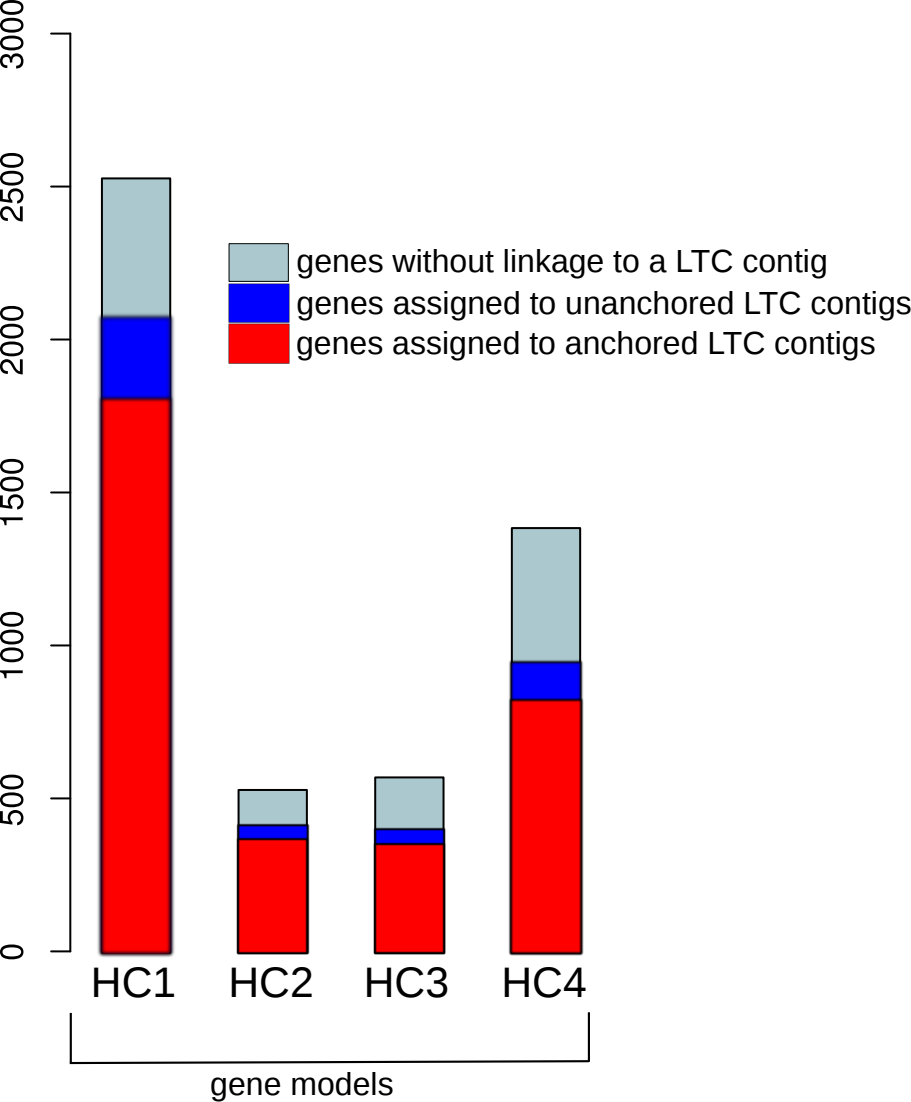

Supplement: Table S3 — Comparison of 1108 ltc-assembled physical contigs of 6AL with fpc at different stringencies. [file tpj0079-0334-SD7.pdf]
