# Supplementary material for: Whole-genome profiling and shotgun sequencing delivers an anchored, gene-decorated, physical map assembly of bread wheat chromosome 6A
Source: Plant J. 2014 May 9;79(2):334–47. doi: 10.1111/tpj.12550 (PMC4241024; doi:10.1111/tpj.12550)
Supplement: Appendix S2 — 6AS ltc-derived physical map. [file tpj0079-0334-SD9.doc]

| **Table S1. Reduction in number of contigs and singleton assembled using FPC as a result of decreasing cut-off value** | | | | |
| --- | --- | --- | --- | --- |
|  | **Short Arm (6AS)** | | **Long Arm (6AL)** | |
| **Stringency cut-off ^(1)^** | **Contig** | **Singleton** | **Contig** | **Singleton** |
| 1e -75 | 1953 | 8102 | 2201 | 8165 |
| 1e -70 | 1786 | 7692 | 2062 | 7727 |
| 1e -65 | 1619 | 7322 | 1924 | 7296 |
| 1e -60 | 1478 | 7021 | 1785 | 6847 |
| 1e -55 | 1349 | 6693 | 1653 | 6399 |
| 1e -50 | 1223 | 6411 | 1507 | 5974 |
| 1e -45 | 1118 | 6167 | 1386 | 5544 |
| 1e -40 | 997 | 5891 | 1228 | 5152 |
| 1e -35 | 914 | 5671 | 1087 | 4757 |
| 1e -30 | 844 | 5494 | 979 | 4337 |
| 1e -25 | 772 | 5333 | 860 | 4077 |
| 1e -20 | 711 | 5217 | 777 | 3845 |
| 1e -15 | 679 | 5118 | 674 | 3664 |
| 1e -11 | 640 | 5045 | 620 | 3560 |
| At each cut-off value, iterative processes of single-to-end and end-to-end were followed by re-building and DQing of the newly formed contigs.  FPC, FingerPrinted Contigs | | | | |
