# Supplementary material for: Whole-genome profiling and shotgun sequencing delivers an anchored, gene-decorated, physical map assembly of bread wheat chromosome 6A
Source: Plant J. 2014 May 9;79(2):334–47. doi: 10.1111/tpj.12550 (PMC4241024; doi:10.1111/tpj.12550)
Supplement: Appendix S3 — 6AL ltc-derived physical map. [file tpj0079-0334-SD10.doc]

| **Table S2. Comparison of 1214 LTC-assembled physical contigs of 6AS with FPC at different stringencies** | | | | | | | | | | |
| --- | --- | --- | --- | --- | --- | --- | --- | --- | --- | --- |
|  | **not found in FPC**^(1)^ | | **LTC=FPC**^(2)^ | | **LTC<FPC**^(3)^ | | **LTC>FPC**^(4)^ | | **LTC≥2 FPC**^(5)^ | |
| **FPC at** | **contig** | **clone/contig** | **contig** | **clone/ contig** | **contig** | **clone/ contig** | **contig** | **clone/ contig** | **contig** | **clone/ contig** |
| 6AS_1e-11 | 539 | 2.6 | 306 | 3.5 | 193 | 25.3 | 104 | 15.5 | 72 | 85.5 |
| 6AS_1e-15 | 544 | 2.6 | 309 | 3.6 | 167 | 23.2 | 118 | 18.1 | 76 | 86.3 |
| 6AS_1e-20 | 548 | 2.7 | 308 | 3.6 | 146 | 21.3 | 125 | 17.5 | 87 | 83.6 |
| 6AS_1e-25 | 549 | 2.7 | 303 | 3.4 | 120 | 17.5 | 136 | 16 | 106 | 79.6 |
| 6AS_1e-30 | 550 | 2.7 | 294 | 3.2 | 92 | 13.8 | 150 | 13.3 | 128 | 74.6 |
| 6AS_1e-35 | 552 | 2.7 | 285 | 3 | 80 | 10.4 | 152 | 10.4 | 145 | 72.3 |
| 6AS_1e-40 | 552 | 2.7 | 278 | 2.9 | 68 | 8.7 | 162 | 9.7 | 154 | 70.3 |
| 6AS_1e-45 | 552 | 2.7 | 271 | 2.6 | 50 | 5 | 167 | 7.9 | 174 | 66.3 |
| 6AS_1e-55 | 557 | 2.7 | 268 | 2.5 | 30 | 4.8 | 168 | 5 | 191 | 63.7 |
| 6AS_1e-60 | 557 | 2.7 | 262 | 2.4 | 27 | 4.9 | 169 | 4.4 | 199 | 61.7 |
| 6AS_1e-65 | 558 | 2.7 | 258 | 2.4 | 22 | 3.5 | 165 | 3.8 | 211 | 59.3 |
| 6AS_1e-70 | 558 | 2.7 | 254 | 2.3 | 17 | 3.4 | 165 | 3.3 | 220 | 57.6 |
| 6AS_1e-75 | 559 | 2.7 | 247 | 2.3 | 13 | 3.2 | 168 | 3 | 227 | 56.1 |
| ^(1)^ not in FPC: there are LTC contigs for which none of the clones were assembled in any contig using FPC tool, ^(2)^ LTC=FPC: all clones of a LTC contig match all clones in FPC, ^(3)^ LTC<FPC: fewer clones in LTC than in FPC, ^(4)^ LTC>FPC: fewer clones in FPC than in LTC. ^(5)^ LTC contigs for which their BACs were assembled into two or more different contigs via FPC.. LTC, Linear Topological Contig; FPC, FingerPrinted Contig | | | | | | | | | | |
