# Supplementary material for: Whole-genome profiling and shotgun sequencing delivers an anchored, gene-decorated, physical map assembly of bread wheat chromosome 6A
Source: Plant J. 2014 May 9;79(2):334–47. doi: 10.1111/tpj.12550 (PMC4241024; doi:10.1111/tpj.12550)
Supplement: Appendix S4 — The fpc-based assembly of the WGP™-based BAC fingerprints. [file tpj0079-0334-SD11.doc]

| **Table S3. Comparison of 1108 LTC-assembled physical contigs of 6AL with FPC at different stringencies** | | | | | | | | | | |
| --- | --- | --- | --- | --- | --- | --- | --- | --- | --- | --- |
|  | **not found in FPC**^(1)^ | | **LTC=FPC**^(2)^ | | **LTC<FPC**^(3)^ | | **LTC>FPC**^(4)^ | | **LTC≥2 FPC**^(5)^ | |
| **FPC at** | **contig** | **clone/contig** | **contig** | **clone/ contig** | **contig** | **clone/ contig** | **contig** | **clone/ contig** | **contig** | **clone/ contig** |
| 6AL_1e-11 | 368 | 2.8 | 139 | 4.5 | 344 | 15.9 | 114 | 17.6 | 143 | 34 |
| 6AL_1e-15 | 372 | 2.8 | 145 | 4.6 | 307 | 14.8 | 127 | 17.6 | 157 | 35.1 |
| 6AL_1e-20 | 373 | 2.8 | 146 | 4.6 | 253 | 13.7 | 147 | 16.9 | 189 | 33.7 |
| 6AL_1e-25 | 376 | 2.9 | 150 | 4.3 | 203 | 12.2 | 173 | 16.4 | 206 | 34 |
| 6AL_1e-30 | 379 | 2.9 | 153 | 4.4 | 159 | 10.8 | 168 | 14 | 249 | 33.3 |
| 6AL_1e-35 | 381 | 2.9 | 152 | 4.2 | 126 | 9.3 | 171 | 12.3 | 278 | 32.9 |
| 6AL_1e-40 | 385 | 2.9 | 151 | 4.2 | 93 | 8.9 | 168 | 9.8 | 311 | 32.1 |
| 6AL_1e-45 | 385 | 2.9 | 140 | 3.5 | 70 | 7.5 | 167 | 7.8 | 346 | 31.2 |
| 6AL_1e-50 | 386 | 2.9 | 136 | 3.4 | 47 | 6.9 | 173 | 6.9 | 366 | 30.2 |
| 6AL_1e-55 | 388 | 2.9 | 130 | 3.2 | 38 | 7 | 172 | 5.5 | 380 | 30.1 |
| 6AL_1e-60 | 389 | 2.9 | 130 | 3.1 | 28 | 6.5 | 164 | 4.8 | 397 | 29.4 |
| 6AL_1e-65 | 389 | 2.9 | 123 | 2.8 | 19 | 5.2 | 165 | 4.3 | 412 | 28.9 |
| 6AL_1e-70 | 389 | 2.9 | 115 | 2.7 | 16 | 4.9 | 166 | 3.9 | 422 | 28.4 |
| 6AL_1e-75 | 389 | 2.9 | 113 | 2.6 | 10 | 4.4 | 163 | 3.5 | 433 | 28 |
| ^(1)^ not in FPC: there are LTC contigs for which none of the clones were assembled in any contig using FPC tool, ^(2)^ LTC=FPC: all clones of a LTC contig match all clones in FPC, ^(3)^ LTC<FPC: more clones in LTC than in FPC, ^(4)^ LTC>FPC: more clones in FPC than in LTC. ^(5)^ LTC contigs for which their BACs were assembled into two or more different contigs via FPC.  LTC, Linear Topological Contig;  FPC, FingerPrinted Contig | | | | | | | | | | |
