# Supplementary material for: Whole-genome profiling and shotgun sequencing delivers an anchored, gene-decorated, physical map assembly of bread wheat chromosome 6A
Source: Plant J. 2014 May 9;79(2):334–47. doi: 10.1111/tpj.12550 (PMC4241024; doi:10.1111/tpj.12550)
Supplement: Appendix S5 — The fpc-based BAC assembly. [file tpj0079-0334-SD12.doc]

**SUPPORTING TEXT:**

**Appendix S1**

**Whole Genome Profiling (WGP^TM^) of 6A chromosome arms**

A total number of 24,576 (64 *x* 384-well plates) and 22,656 (59 *x* 384-well plates) BACs of the short and long arm, respectively, served as input for WGP^TM^ (performed at Keygene NV, Wageningen, The Netherlands, [http://keygene.com](http://keygene.com/)) (Table 1). The WGP methodology was conducted as described by Van Oeveren et al (van Oeveren et al., 2011). The entire two libraries were separately 3D (3-Dimensional) pooled (see Material & Methods) and then subjected to digestion by a combination of *Hind*III and *Mse*I restriction enzymes, followed by ligation with barcoded adaptors. Subsequent sequencing of the respective fragments from the *Hind*III restriction site yielded 99.8 and 165.4 million reads for short and long arm, respectively (Table 2), with a 100 nt read length. The reads had to contain a valid sample identification tag and proper restriction site sequence. Tag deconvolution (using the first 50 nt of the 100 nt reads) finally eventuated in almost 50% of the total number of reads per chromosome arm being assigned as WGP tags to individual BACs. The 50 nt was previously identified being the most optimal tag length (Sierro et al., 2013). After filtering the generated WGP tags using several quality criteria (see Material & Methods), a total of 109,611 non-redundant WGP tags mapped to the 19,289 short arm BACs (85.0% of the initial library) and 108,811 non-redundant WGP tags were mapped to 18,660 BACs of the long arm (75.9% of the initial library) (Table 2). This delivered on average 29.2 WGP tags per BAC for 6AS and 27 WGP tags per BAC for 6AL by considering all BACs for a given WGP tag (Table 2). BACs without any tag could be the result of a small insert, 'empty' BACs, technical bottlenecks during pooling and sequencing and/or possibly presence of overlapping BACs from the same region (Philippe et al., 2012).
