# Supplementary material for: Whole-genome profiling and shotgun sequencing delivers an anchored, gene-decorated, physical map assembly of bread wheat chromosome 6A
Source: Plant J. 2014 May 9;79(2):334–47. doi: 10.1111/tpj.12550 (PMC4241024; doi:10.1111/tpj.12550)
Supplement: Appendix S8 — 6A ltc contigs and the associated sequence information. [file tpj0079-0334-SD15.doc]

**SUPPORTING EXPERIMENTAL PROCEDURES:**

**Appendix S4**

**The FPC-based assembly of the WGP-based BAC fingerprints**

Contig assembly was first performed using the FingerPrinted Contig (FPC) program (v9.4; <http://www.agcol.arizona.edu/software/fpc/>). The WGP tags were adapted for usage in FPC by converting each unique sequence tag into a number (to create the respective size file) (van Oeveren et al., 2011). This adaptation generated pseudo restriction fragment sizes for which the FPC software was originally developed. Because the WGP tags are uniquely defined by their sequence composition, FPC could be used at the highest stringency setting—the tolerance value of 0. The BAC assemblies were performed using the methodology described for the construction of the WGP-based physical map of chromosome 3B (Philippe et al., 2012). In brief, the initial FPC assembly was performed with a cut-off value of 1e-75. This was subsequently run through single-to-end and end-to-end merging (Match: 1, FromEnd: 13) at 13 sequentially higher cut-off (i.e. lower stringency) that ended up at 1e-11 as was suggested for WGP based strategy in wheat (Philippe et al., 2012). The DQing function was used at each cut-off value to break up all contigs that contained more than 10% of Questionable (Q) clones. This was then followed by rebuilding the contig at the same cut-off value of the corresponding step. The remaining FPC parameters were set as gel length of 110000 and the band sizes of 4700 nt.
