# Supplementary material for: Whole-genome profiling and shotgun sequencing delivers an anchored, gene-decorated, physical map assembly of bread wheat chromosome 6A
Source: Plant J. 2014 May 9;79(2):334–47. doi: 10.1111/tpj.12550 (PMC4241024; doi:10.1111/tpj.12550)
Supplement: Appendix S9 — 6A ltc-derived contigs genetically anchored to the genetic maps of wheat. [file tpj0079-0334-SD16.doc]

**Appendix S5**

**The FPC-based BAC assembly**

We also operated the BAC assembly using the FPC software accustomed for use with WGP tags (van Oeveren et al., 2011). The FPC assemblies were built using the same set of BACs and WGP tags that were employed for LTC analysis. For both of the arms, only BAC clones containing 6 to 68 tags entered to the BAC assembly pipeline. A total of 18,820 BAC clones and 109,570 unique WGP tags were used as input for the BAC assembly of 6AS (Table 1). In case of 6AL, the number of BACs utilized to build the physical map amounted to 17,309 clones that contained a total number of 108,700 unique WGP tags (Table 1). The FPC assembly started at a cut-off value of 1e-75 resulting in 1,953 and 2,201 FPC contigs for the short and long arm, respectively. At this step, a total number of 8,102 BACs (6AS) and 8,165 BACs (6AL) did not link to any other BAC, and thus, were automatically classified as singletons (Table S1). We then further performed 13 singleton-to-end and end-to-end contig merging steps of FPC assembly until the final cut-off value of 1e-11 was reached as recommended by Philippe et al. (Philippe et al., 2012) for WGP-based physical map formation in wheat. We observed a constant reduction in the number of both FPC contigs and singletons during this consecutive decline in the assembly stringency (Table S1). The final assembly was achieved by a total number of 640 FPC contigs for 6AS and 620 contigs for 6AL. A total number of 5,045 and 3,560 BACs were sorted out as singletons for 6AS and 6AL, respectively (Table S1, Data S6, and Data S7). For 6AS the chromosome arm size was estimated of being 451.8 Mb with an average contig size of 0.706 Mb and L50 contig size equal to 2.2 Mb. The 6AL FPC contig assembly was gauged with a size of 483.5 Mb, an average contig size of 0.780 Mb Mb and L50 contig size of 1.5 Mb. These calculations were made after multiplying FPC band units by the average distance of 4,700 nt between two consecutive WGP tags. The latter was estimated by dividing the average BAC insert size by the average number of WGP tags per BAC.
