# Supplementary material for: Whole-genome profiling and shotgun sequencing delivers an anchored, gene-decorated, physical map assembly of bread wheat chromosome 6A
Source: Plant J. 2014 May 9;79(2):334–47. doi: 10.1111/tpj.12550 (PMC4241024; doi:10.1111/tpj.12550)
Supplement: Appendix S10 — 6A ltc-derived contigs anchored to barley genomic resources. [file tpj0079-0334-SD17.doc]

**AppendixS6.**

**Construction of an *in silico*-anchored, gene-decorated physical map**

To execute an efficient sequence homology search against wheat genetic markers with known sequences, WGP sequence tags were extended by connecting them to three resources of publicly available wheat or wheat genome related sequence information (Figure S1). These contained bread wheat chromosome 6A arm-specific shotgun sequence contigs (WCS) obtained from flow-sorted 6A chromosome arms (IWGSC; <http://www.wheatgenome.org/>) and sequences from the wheat progenitor genomes, including *T. urartu* (Ling et al., 2013) and *Ae. tauschii* (Jia et al., 2013). The Vmatch tool ([www.vmatch.de](http://www.vmatch.de/)) was used to perform the respective sequence homology search allowing zero nucleotide error matches over the length of the corresponding WGP sequence tags, when tag connected to WCS contigs. Then, *T. urartu* and *Ae. tauschii* sequences were added only to the already assigned 6A WCS contigs, allowing only two nucleotide error matches requiring a match length of at least 200 nucleotides. All sequence information underlying the physical contigs were then employed for sequence homology searches against all available genetic markers with known sequence via the aforementioned Vmatch tool and homology search criteria with zero nucleotide error. In the current study, we utilized genetic markers derived from two different available genetic maps reported by Poland et al. (2012), and Cavanagh et al. (2013). LTC contigs were anchored separately per marker map and then combined to form a merged marker backbone. In short, an already reported approach (IBSC, 2012) has been applied with small modification through which marker sequences from both wheat maps (Cavanagh et al., 2013 and Poland et al., 2012 maps) were independently mapped on physical contigs associated sequences using Vmatch (<http://www.vmatch.de/>). For each marker, the best match to a physical contig was taken requiring a match length of at least 50 nt. Next, a median genetic position (cM) of all matching markers was used to assign the corresponding physical contigs to a genetic position. This procedure was applied for both marker maps separately. For establishment of the integrated genetic map, first physical contigs received a final genetic position by first considering the genetic position derived from the Cavanagh et al., (2013) map. This map is transcript-derived, originated from different wheat cultivars, and has been considered as a reliable genetic map compared to the GBS map by Poland et al. (2012). For contigs with no marker from the Cavanagh et al. (2013) map, markers from the GBS map (Poland et al., 2012) were considered and similarly a cM median value was calculated for the respective contigs. The resulting values were then interpolated to a cM position of the Cavanagh et al. (2013) map using linear regression. To compensate for the different cumulative map length in the two maps, the resulting GBS genetic position was multiplied by the factor 1.2. All falsely anchored contigs (6AL contigs anchored to 6AS and vice versa) were then removed. We further extended the anchoring efforts by considering two of the barley genetically anchored sequence resources. These included 15,719 high-confidence barley genes from the barley genome (IBSC, 2012) together with 723,499 anchored WGS (Whole Genome Shotgun) contigs from the barley POPSEQ data (Mascher et al., 2013). POPSEQ WGS sequences were assigned to the physical map of 6A using Vmatch when satisfied a best bi-directional hit with the following parameter of seed length=20, exdrop=1 and identity>=87% and required length of 300 nt. For the mapping of high-confidence barley genes the following parameters were used: seed length=12, exdrop=2, identity>=87% and hit length of at least 100 nt. For mapping against other reference genomes, Vmatch parameters including seed length=20, exdrop 2 and identity>=75% was used, only best bi-directional hits were considered.
