# Supplementary material for: Whole-genome profiling and shotgun sequencing delivers an anchored, gene-decorated, physical map assembly of bread wheat chromosome 6A
Source: Plant J. 2014 May 9;79(2):334–47. doi: 10.1111/tpj.12550 (PMC4241024; doi:10.1111/tpj.12550)
Supplement: Appendix S11 — An estimation of the recombination frequencies along the chromosome. [file tpj0079-0334-SD18.doc]

**AppendixS7**

**The effect of incorporating *Ae. tauschii* sequences to the overall anchoring of the 6A physical map**

*Ae. tauschii* sequences were added to the already assigned 6A WCS contigs using highly stringent sequence homology search criteria that allowed two nucleotide mismatches over at least 200 nucleotides match length (Methods S2). We then checked how inclusion of *Ae. tauschii* sequence resources would affect the genetic integration of the corresponding physical contigs compared to the anchoring without *Ae. tauschii*. Therefore, we performed an anchoring of the physical contigs with and without considering *Ae. tauschii* sequence information using the Cavanagh et al. (2013) map alone. We observed a correlation coefficient of 0.999 between genetic anchoring using *Ae. tauschii* dataset and without it. This indicates that connecting *Ae. tauschii* sequences to the physical map has no negative effect on the accuracy of the anchoring analysis of the physical map. Moreover, we observed that incorporating *Ae. tauschii* sequences has a limited contribution to the overall anchoring of the physical map, at least when the Cavanagh et al. (2013) map was considered. This could be concluded because the amount of physical map being anchored to the respective genetic map was only reduced by 23 Mb from 183 Mb to 160 Mb without *Ae. tauschii* sequences.
