# Supplementary material for: Whole-genome profiling and shotgun sequencing delivers an anchored, gene-decorated, physical map assembly of bread wheat chromosome 6A
Source: Plant J. 2014 May 9;79(2):334–47. doi: 10.1111/tpj.12550 (PMC4241024; doi:10.1111/tpj.12550)
Supplement: Appendix S15 — Scaffolding the ltc contig. [file tpj0079-0334-SD22.doc]

**AppendixS11.**

**An estimation of the recombination frequencies along the chromosome**

The large portion of the physical map anchored to the respective wheat genetic maps allowed us to analyze recombination frequencies along the entire wheat 6A chromosome. To this end, the underlying integrated genetic map was divided into 28 bins, each 10 cM in size. The physical map was anchored to each of the bins to measure the pattern of recombination along the chromosome (Figure 2). As expected for plants with large genomes such as maize (Anderson et al., 2003), barley (Kunzel et al., 2000) and wheat (Lukaszewski and Curtis, 1993), we were able to reconfirm that recombination has followed a particular pattern with a steep increase from the centromere towards telomeres. The centromeric area showed a very low level of recombination, where almost 142 Mb (or 21% of the anchored proportion of the physical map) of the physical map was allocated to a single bin of 10 cM. This also indicates the limitation in our genetic anchoring analysis by which a high number of physical contigs are anchored to the (peri-) centromeric area with yet unclear order. In contrast, the terminal bin of 6AL showed the highest rate of recombination on this chromosome arm even though only 0.3 Mb in size. Moreover, we frequently observed oscillating changes of recombination frequencies along the chromosome arms, which is fairly in line with previous findings in wheat (Erayman et al., 2004) and other related genomes like barley (Kunzel et al., 2000; IBSC, 2012). These detailed estimates of recombination frequencies along wheat chromosome 6A will be most useful for future map-based cloning and gene identification projects.
