# Supplementary material for: Whole-genome profiling and shotgun sequencing delivers an anchored, gene-decorated, physical map assembly of bread wheat chromosome 6A
Source: Plant J. 2014 May 9;79(2):334–47. doi: 10.1111/tpj.12550 (PMC4241024; doi:10.1111/tpj.12550)
Supplement: Appendix S16 — 6A ltc-derived contigs mergable via Tu contigs (ltc scaffolds). [file tpj0079-0334-SD23.doc]

**AppendixS12.**

**Complementary description of five different classes of LTC contigs when compared with individual assemblies obtained via FPC.**

Comparing contigs obtained by LTC (as reference assembly; Figure 3) with individual assemblies obtained via FPC (Table S1, Appendix S1) at different stringencies revealed five different classes of LTC contigs. These groups include:

1. LTC-specific contigs (not found in FPC)
2. LTC contigs that comprised the same BAC order and composition (LTC=FPC)
3. longer (LTC>FPC) than their counterparts in FPC
4. LTC contigs that were shorter (LTC<FPC)
5. those LTC contigs for which their BACs were assembled into two or more different contigs via FPC (LTC≥2FPC; flagged as cases of conflicts FPC/LTCs) (Figure 3, Table S2 & Table S3),.

While the three of the five groups of LTC contigs (group 1, 2 and 3) did not dramatically change in number across the FPC assemblies, the number of contigs in the other two groups, including LTC<FPC (4) and LTC≥2FPC (5), showed significant variation. Such changes were observed while decreasing the assembly cut-off value in FPC (Figure S3). First, a sharp increase in the number of FPC contigs that were longer than their LTC analogs (LTC<FPC) was observed, and secondly, cases of conflicts between the two tools declined (Figure S3). Both patterns observed were mainly explainable by singleton-to- end and end-to-end merging operations during FPC assembly (see Discussion for more details). Moreover, the three groups of contigs, including LTC=FPC, LTC>FPC and LTC<FPC, that comprised almost 50% of the LTC assembly revealed either 100% similarity (LTC=FPC) between FPC and LTC in detecting BAC overlaps, or the difference between LTC contigs and the corresponding FPC contigs could be explained by extending contigs with more BACs either via LTC (in case of LTC>FPC) or FPC (in case of LTC<FPC). In the latter case, shared BACs kept the relatively same order in contigs assembled by both tools.
