# Supplementary material for: Whole-genome profiling and shotgun sequencing delivers an anchored, gene-decorated, physical map assembly of bread wheat chromosome 6A
Source: Plant J. 2014 May 9;79(2):334–47. doi: 10.1111/tpj.12550 (PMC4241024; doi:10.1111/tpj.12550)
Supplement: Supplementary file 26 [file tpj0079-0334-SD26.doc]

**AppendixS15.**

**Scaffolding the LTC contig**

We utilized the available sequence scaffolds from *T. urartu* to check whether they allowed to bridge LTC contigs. To this end, it was essential to first order the corresponding WGP tags along the physical contigs. Therefore, WGP tags underlying the LTC contigs were bin-mapped and ordered according to their CB (Consensus Band) unit along the corresponding LTC contig as described by (Sierro et al., 2013). The bin window was defined per average clone position (start+end)/2, the corresponding WGP tags were counted only once so that each tag was assigned uniquely to a single bin. These WGP tags were then mapped to the publicly available *T. urartu* whole genome shotgun sequence contigs (Tu contigs). Next, we searched for LTC contig scaffolds, in that, if a single Tu contig matched at least three tags from terminal bins (including first/last bins) of exclusively only two different LTC contigs. To increase scaffolding efficiency only a portion of the longer sized Tu sequences, named as Tu scaffolds (L50 of 64,532 nt), were used (in total 90.7 Mb) (Jia et al., 2013). Sequence match for a given bin required WGP tag length-equal direct homology with a single nt mis-match allowed.
