# Supplementary material for: Whole-genome profiling and shotgun sequencing delivers an anchored, gene-decorated, physical map assembly of bread wheat chromosome 6A
Source: Plant J. 2014 May 9;79(2):334–47. doi: 10.1111/tpj.12550 (PMC4241024; doi:10.1111/tpj.12550)
Supplement: Supplementary file 28 [file tpj0079-0334-SD28.doc]

**AppendixS17**

**BAC Pooling Scheme for WGP data production**

A 3-dimensional (3D) format of BAC pools was made for each of the BAC libraries. For each 384-well plate, the 3D pooling format comprised pools with two rows (48 BACs each), pools with three columns (48 BACs each) and an additional set of pools to differentiate the group of six BACs labeled by a single row and column pool combination (termed split-box pools with 64 BACs each) was manufactured. This resulted for each 384-well plate pool of BAC clones in 8 row pools, 8 column pools and 6 split-box pools. This allowed four 384-well plates to fill 88 pooled wells in a single 96-well plate. Pooled BACs were subsequently subjected for isolation of high-concentration BAC DNA, followed by WGP sample preparation as previously described (van Oeveren et al., 2011).
