# Supplementary material for: Whole-genome profiling and shotgun sequencing delivers an anchored, gene-decorated, physical map assembly of bread wheat chromosome 6A
Source: Plant J. 2014 May 9;79(2):334–47. doi: 10.1111/tpj.12550 (PMC4241024; doi:10.1111/tpj.12550)
Supplement: Supplementary file 29 [file tpj0079-0334-SD29.doc]

**SUPPORTING INFORMATION**

**DATA AVAILABILITY:**

The URL to view the wheat 6A physical map, to blast against assigned sequence information and to download the respective sequences:

<http://seacow.helmholtz-muenchen.de/cgi-bin/gb2/gbrowse/Wheat_PhysMap_6A>

**SUPPORTING FIGURES:**

**Figure S1.** Steps for the elongation of WGP tags by connecting them to the available 6A related sequence information. These in silico connections were performed to facilitate anchoring of BAC contigs to the corresponding wheat genetic markers. Sequences include 6A Whole Chromosome Sequence contigs (WCS) (IWGSC; <http://www.wheatgenome.org/>), whole genome assembly of *Triticum urartu* (Tu contigs) (Ling et al., 2013), and *Aegilops tauschii* (Ae contigs) (Jia et al., 2013).

**Figure S2.** Different amount of publicly available sequence information was connected to the physical contigs using the underlying WGP tags. Sequences include 6A Whole Chromosome Sequence contigs (WCS) (IWGSC; <http://www.wheatgenome.org/>), whole genome assembly of *Triticum urartu* (Tu contigs) (Ling et al., 2013), and *Aegilops tauschii* (Ae contigs) (Jia et al., 2013).

**Figure S3.** An example of homology between LTC and FPC contigs (as reference). LTC contigs were aligned against FPC contigs at a cut-off value 1e-50. The aim was to test whether the gaps in LTC-assembled contigs can be filled using FPC-assembled contigs. BACs were arranged on the basis of their position (CB units) along the corresponding contigs. Common BACs between LTC and FPC assemblies are shown in green and connected by lines.

**Figure S4.** Different gene classes assigned to the physical contig. The 6A WCS contigs underlying the gene sequences were connected to the physical contigs using WGP tags. WCS gene annotation and the gene classification was performed by IWGSC (IWGSC, 2014; In review). Classification was performed on level of similarity compared with the reference genes from *Brachypodium*, rice and sorghum. HC1 are class of genes with more than70% overlap in the coding sequence. HC2 are those genes having 50 to less than 70% , HC3 with 30 to less than 50% , and HC4 are all below 30% similarity (IWGSC, 2014, under review).
