# Supplementary material for: Whole-genome profiling and shotgun sequencing delivers an anchored, gene-decorated, physical map assembly of bread wheat chromosome 6A
Source: Plant J. 2014 May 9;79(2):334–47. doi: 10.1111/tpj.12550 (PMC4241024; doi:10.1111/tpj.12550)
Supplement: Supplementary file 30 [file tpj0079-0334-SD30.doc]

**SUPPORTING DATA:**

**Appendix S2.**  6AS LTC-derived physical map: LTC-assembled contigs including contig IDs, corresponding BACs, BAC order and position as well as BACs selected for MTP.

**Appendix S3.**  6AL LTC-derived physical map: LTC assembled contigs including contig IDs, corresponding BACs, BAC order and position as well as BACs selected for MTP.

**Appendix S8.** 6A LTC contigs and the associated sequence information: WGP underlying the LTC contigs were elongated via 6A Whole Chromosome Sequence contigs (6A WCS), whole genome assembly of *T. urartu*, and *Ae. tauschii*. The LTC contigs IDs and all corresponding sequence IDs are provided.

**Appendix S9.** 6A LTC-derived contigs genetically anchored to the genetic map of wheat: Wheat genetic maps of Cavanagh et al. (2013) and Poland et al., (2012) as well as the integrated map of the two (constructed in the current study; Methods S2) were used.

**Appendix S10.** 6A LTC-derived contigs anchored to barley genomic resources. Barley resources used for anchoring included 15,719 high-confidence barley genes from the barley genome (IBSC, 2012), together with 723,499 anchored WCS contigs from barley POPSEQ data (Mascher et al., 2013).

**Appendix S13.** 6AS final FPC-derived physical map at1e-11.xls: Contains final FPC-based assembly of the chromosome arm including contig IDs, corresponding BACs, BAC order and position

**Appendix S14.** 6AL final FPC-derived physical map at1e-11.xls: Contains final FPC-based assembly of the chromosome arm including contig IDs, corresponding BACs, BAC order and position.

**Appendix S16.** 6A LTC-derived contigs mergable via Tu contigs (LTC scaffolds): Contains potential mergable LTC contigs for which a Tu contig matched at least three tags from terminal bins (including first/last bins) of exclusively two different LTC contigs.
